# Prognostic value of lymphovascular invasion in patients with squamous cell carcinoma of the penis following surgery

**DOI:** 10.1186/s12885-019-5714-1

**Published:** 2019-05-21

**Authors:** Kai Li, Jian Sun, Xuedong Wei, Guang Wu, Fei Wang, Caibin Fan, Hexing Yuan

**Affiliations:** 1 Department of Urology, Suzhou Municipal Hospital, 26 Daoqian Road, Suzhou, 215000 Jiangsu Province, People’s Republic of China; 2 Department of Urology, First Affiliated Hospital of Soochow University, 188 Shizi Road, Suzhou, 215000 Jiangsu Province, People’s Republic of China

**Keywords:** Lymphovascular invasion, Prognosis, Squamous cell carcinoma of the penis, Survival

## Abstract

**Background:**

To evaluate the prognostic value of Lymphovascular Invasion (LVI) in patients with squamous cell carcinoma of the penis (SCCP) following surgery.

**Patients and methods:**

This retrospective study analyzed the data of 891 eligible patients with SCCP who were diagnosed between 2010 and 2014, obtained from the Surveillance, Epidemiology, and End Results (SEER) database. The patients were categorized by LVI, age, grade, T stage, lymph nodes status, distant metastasis, regional lymph nodes removed, and surgery. Overall survival (OS) and penile carcinoma-specific survival (PCSS) were evaluated by Kaplan-Meier method and Cox proportional hazards regression model.

**Results:**

The presence of LVI was significantly associated with increased risk of advanced T stage, high grade, lymph node metastasis, and distant metastasis (*P* < 0.001 for all). In Kaplan-Meier analyses, patients with the presence of LVI had significantly lower OS and PCSS than those with the absence of LVI (*P* < 0.001 for both,). The presence of LVI was also significantly associated with poorer OS and worse PCSS in patients with Tx + Ta + T1 stage (*P* = 0.007, *P* < 0.001), N0 stage (P < 0.001, *P* = 0.040), grade 1 (*P* = 0.001, P < 0.001), grade 2 (P = 0.001, *P* = 0.014), no distant metastasis (P < 0.001 for both), no regional lymph nodes removed (P < 0.001 for both), Non-radical surgery (P < 0.001 for both) and radical surgery(*P* = 0.037, *P* = 0.002). In multivariate analyses, the presence of LVI in patients with SCCP following surgery was found to be a significant independent predictor of decreased OS (hazard ratio 1.403, *P* = 0.039).

**Conclusions:**

The LVI status might be a crucial prognostic indicator for overall survival in patients with SCCP.

## Background

Penile carcinoma is a rare malignant disease, with an estimated annual incidence of 2–4 cases per 100,000 men in the developing countries of Africa, Asia, and South America; however, it is significantly rarer in the United States and Europe (0.3–1 cases per 100,000 males) [1]. The squamous cell carcinoma of the penis (SCCP) represents over 95% of penile carcinomas and is the most common type [2, 3]. It most commonly occurs at an advanced age; with the peak age of presentation is between 50 and 70 years [3]. Organ preservation strategy is a preferred treatment modality for early stage penile carcinoma; however, surgical resection with a partial or radical penectomy remains the oncological gold standard therapy for advanced invasive penile carcinoma [4].

Lymphovascular invasion (LVI) is described as the presence of tumor cell invasion into blood vessels or the lymphatic system [5]. LVI is a primary and crucial phase in the systemic metastasis of cancer cells [6]. Increasing evidence suggests that the presence of LVI is a poor prognostic indicator in various types of malignancies, including bladder cancer [7], prostate cancer [8], clear cell renal cell carcinoma [9], esophageal cancer [10], breast cancer [5] and lung cancer [11]. Presently, there are only limited studies on the association of LVI with the clinical outcome of patients with penile carcinoma. Recently, evidence showed that the presence of LVI was a significant risk factor for occult micrometastases in patients with penile carcinoma [12]. Moreover, previous studies suggested that lymphovascular embolization was a significant risk factor for increased lymphatic metastasis in patients with penile carcinoma [13]. However, current indication remains elusive. Furthermore, LVI is not considered in guidelines for treatment strategies for penile carcinoma, due to the paucity of adequate data to substantiate the effects of LVI on clinicopathological characteristics and survival outcome. Therefore, the present study aimed to evaluate the association of LVI with clinicopathological characteristics. Further, the impact of LVI on survival outcome of men with SCCP was also investigated.

## Patients and methods

This retrospective study analyzed the data of 891 eligible patients with penile SCCP diagnosed and underwent surgery between 2010 and 2014, obtained from the Surveillance, Epidemiology, and End Results (SEER) database. The database accession number is 13638-Nov2017. According to the “International Classification of Diseases-Oncology, 3rd edition” (ICD-O-3), tumors with codes 8051–8052 and 8070–8075 were classified as pure squamous cell carcinoma [14]. However, cases with incomplete records on LVI, grade, regional lymph nodes removed, and surgery were excluded from the study.

Demographic characteristics of patients including age (< 50 and ≥ 50 years old) and clinicopathological characteristics including LVI, T stage, grade (grade I- IV), lymph nodes status, distant metastasis, regional lymph nodes removed, and surgery (radical surgery and Non-radical surgery) were collected. TNM stages of the penile tumor were determined according to the American Joint Committee on Cancer (AJCC) 7th edition staging system using available clinical and pathologic data on tumor invasion, lymph nodes status, and distant metastasis, respectively. The histopathological grading of penile carcinoma was determined according to the Broder’s classification system [15]. All the specimens were subjected for routine histopathologic examination.

For survival outcome, overall survival (OS) was defined as a time period between the date of SCCP diagnosis and the date of death or last follow-up. Penile carcinoma-specific survival (PCSS), a time period from the date of SCCP diagnosis to the date of carcinoma-specific death or censoring was also determined. The cause of death was obtained from the death certificate. All patients with SCCP were followed up until December 31, 2014, in this study, with a median follow-up period of 16 months (range, 0 to 59).

All statistical analyses were performed using SPSS version 17.0 software (IBM, Chicago, IL, USA). A two-tailed chi-square test was used to determine the significance of differences between categorical variables. The Kaplan-Meier method was used to calculate survival functions, and differences were assessed using the log-rank statistic. Univariate and multivariate analyses were performed using backward stepwise Cox proportional hazards regression model to determine potential prognostic factors for OS and PCSS. Besides, adjusted hazard ratio (HR) and 95% confidence intervals (CI) were used to express the magnitudes of statistical significance in the model. All reported *p-*values were two-sided, and a *p*-value of < 0.05 was considered statistically significant.

## Results

A total of 891 eligible patients with SCCP were included in this study**.** The presence of LVI was detected in 186 patients (20.9%), and 705 patients (79.1%) had SCCP without LVI. The median follow-up time was 16 months (range, 0 to 59). A total of 235 (26.4%) patients with SCCP died during this study. Of the 235 deaths, 91 patients died from SCCP.

The association of LVI with demographic characteristics and clinicopathological characteristics were presented in Table 1. The presence of LVI significantly increased the risk of advanced T stage, high grade, lymph node metastasis, and distant metastasis (*P* < 0.001 for all). However, there was no statistically significant difference in age between the patients with LVI and those without LVI (*P* = 0.125). Besides, patients who removed regional lymph nodes exhibited a lower incidence of SCCP with LVI than those who did not remove regional lymph nodes (*P* < 0.001). Similarly, patients who received radical surgery showed a lower incidence of SCCP with LVI compared to those who did not (*P* = 0.004).

**Table 1:** Association of LVI with clinicopathological characteristics

N(%) variables	All patients	LVI absent	LVI present	*P*
No. of Patients	891	705(79.1)	186(20.9)	
Age				0.125
< 50	100(11.2)	85(85.0)	15(15.0)	
≥ 50	791(88.8)	620(78.4)	171(21.6)	
T stage				**< 0.001**
Tx + Ta + T1	457(51.3)	416(91.0)	41(9.0)	
T2	242(27.2)	186(76.9)	56(23.1)	
T3	178(20.0)	94(52.8)	84(47.2)	
T4	14(1.6)	9(64.3)	5(35.7)	
Lymph nodes status				**< 0.001**
Nx	21(2.4)	13(61.9)	8(38.1)	
N0	704(79.0)	595(84.5)	109(15.5)	
N1-N3	166(18.6)	97(58.4)	69(41.6)	
Grade				**< 0.001**
G1	242(27.2)	222(91.7)	20(8.3)	
G2	462(51.9)	372(80.5)	90(19.5)	
G3 + G4	187(21.0)	111(59.4)	76(40.6)	
Distant metastasis				**< 0.001**
No	864(97.0)	691(80.0)	173(20.0)	
Yes	27(3.0)	14(51.9)	13(48.1)	
Regional lymph nodes removed				**< 0.001**
No	683(76.7)	572(83.7)	111(16.3)	
Yes	208(23.3)	133(63.9)	75(36.1)	
Surgery				**0.004**
Non-radical surgery	853(95.7)	682(80.0)	171(20.0)	
Radical surgery	38(4.3)	23(60.5)	15(39.5)	

*LVI* lymphovascular invasion; *SCCP* squamous cell carcinoma of the penis

Significant values in bold

**Fig. 1:**
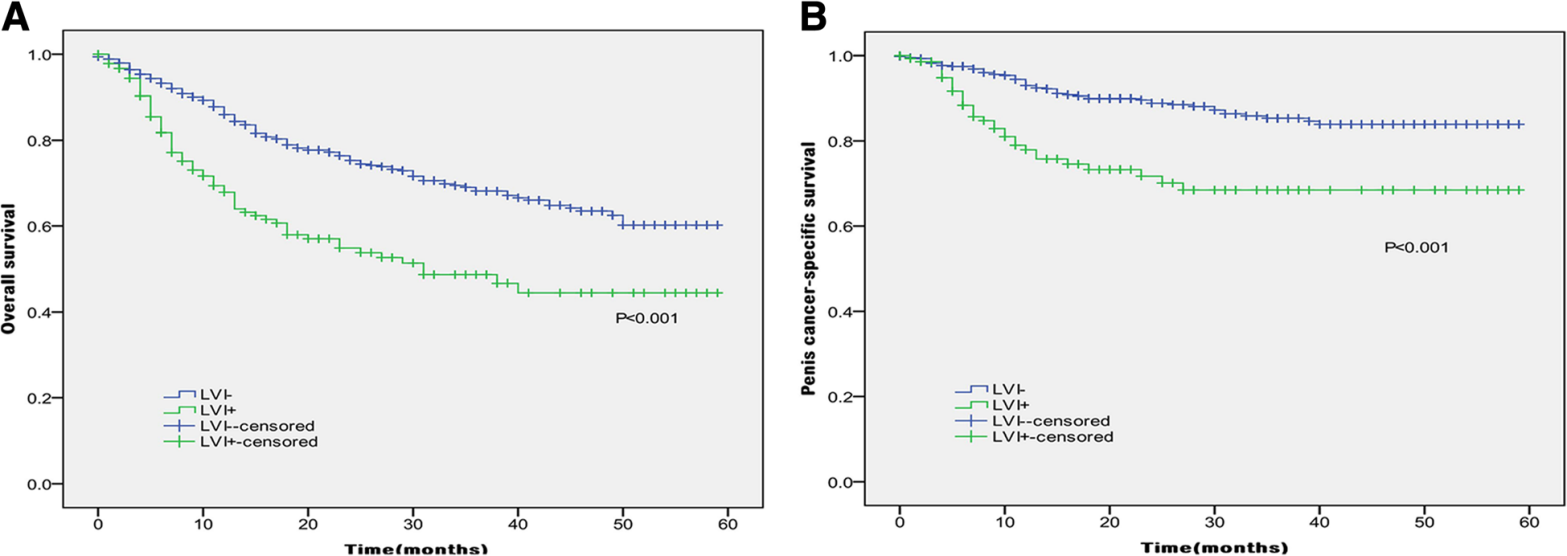
Kaplan-Meier analyses of overall survival (**a**) and penile carcinoma-specific survival (**b**) in 891 patients treated with surgery stratified by LVI status.

**Fig. 2:**
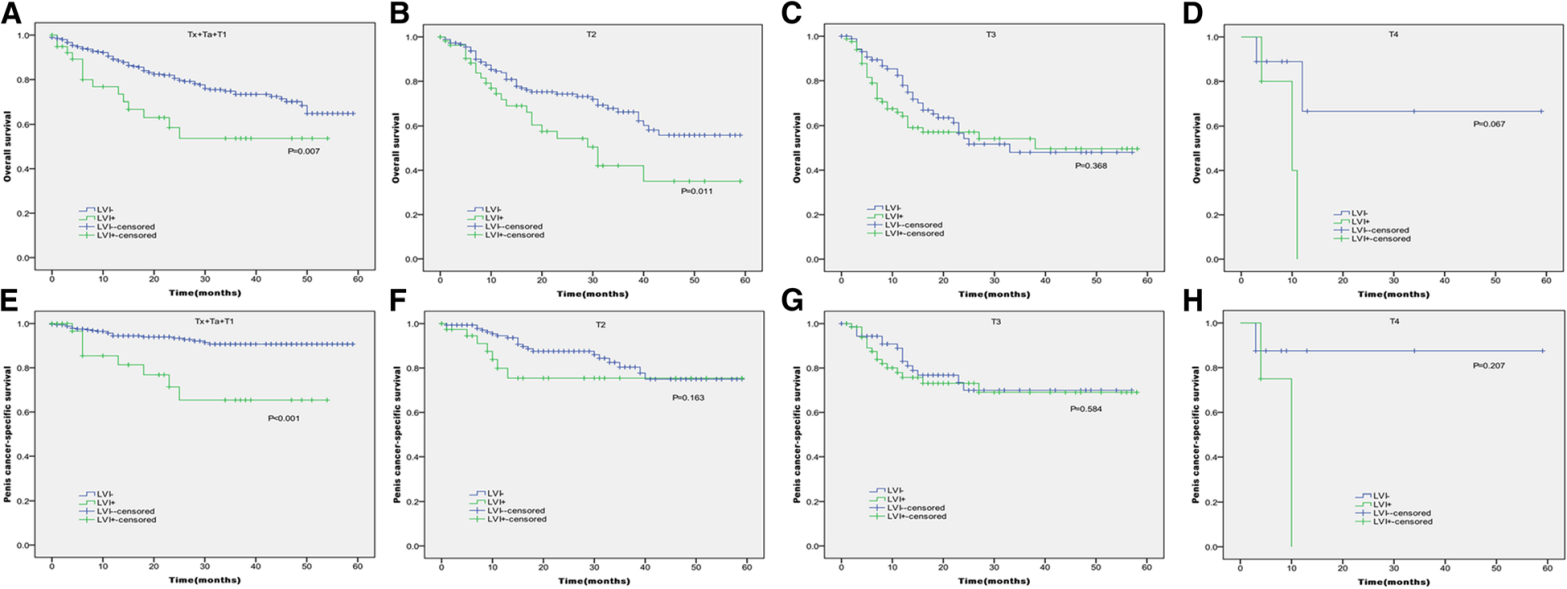
Kaplan-Meier analyses of overall survival (**a**, **b**, **c**, **d**) and penile carcinoma-specific survival (**e**, **f**, **g**, **h**) within each T stage in patients treated with surgery stratified by LVI status

**Fig. 3:**
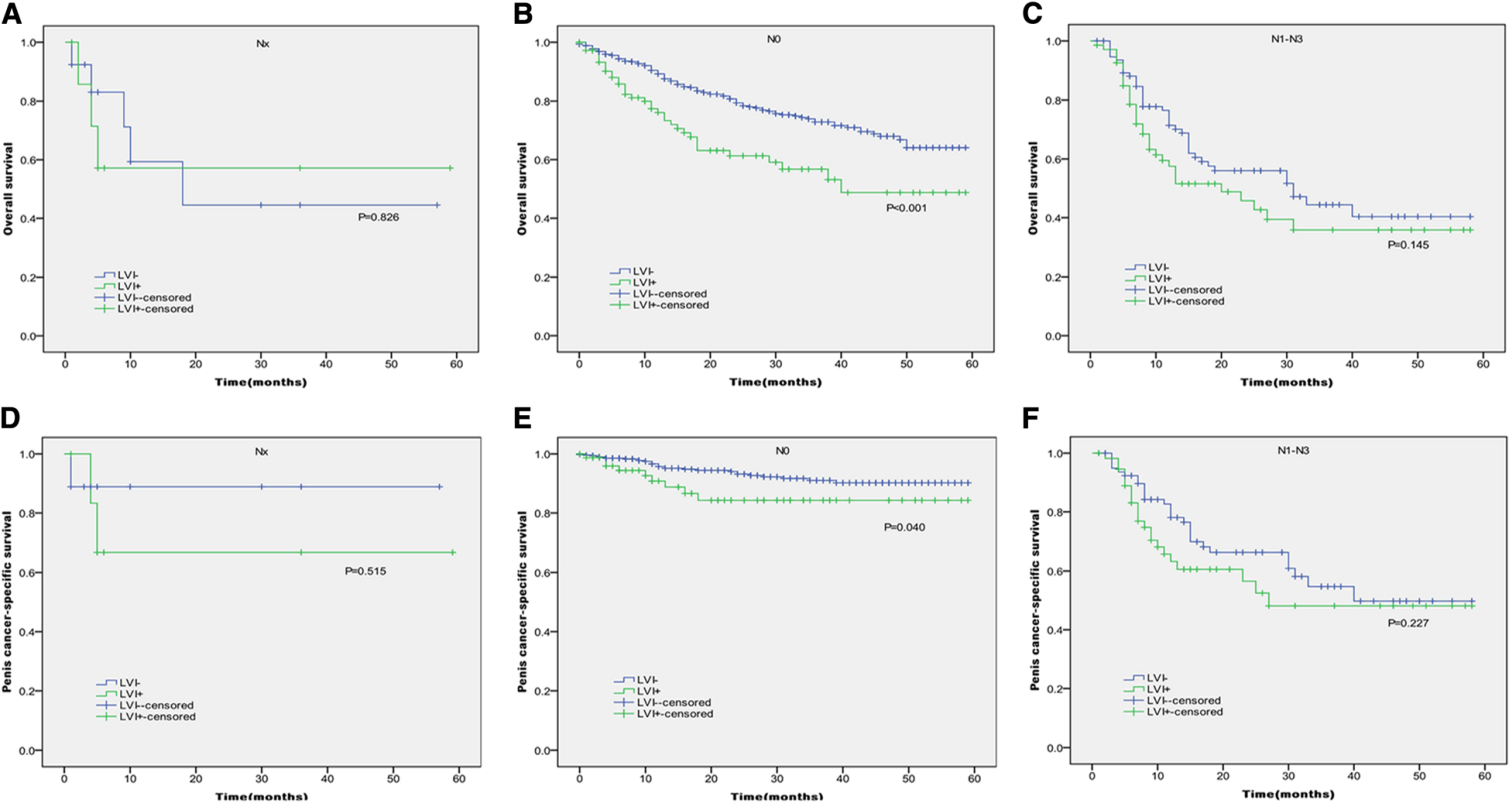
Kaplan-Meier analyses of overall survival (**a**, **b**, **c**) and penile carcinoma-specific survival(**d**, **e**, **f**) within each lymph nodes status in patients treated with surgery stratified by LVI status

**Fig. 4:**
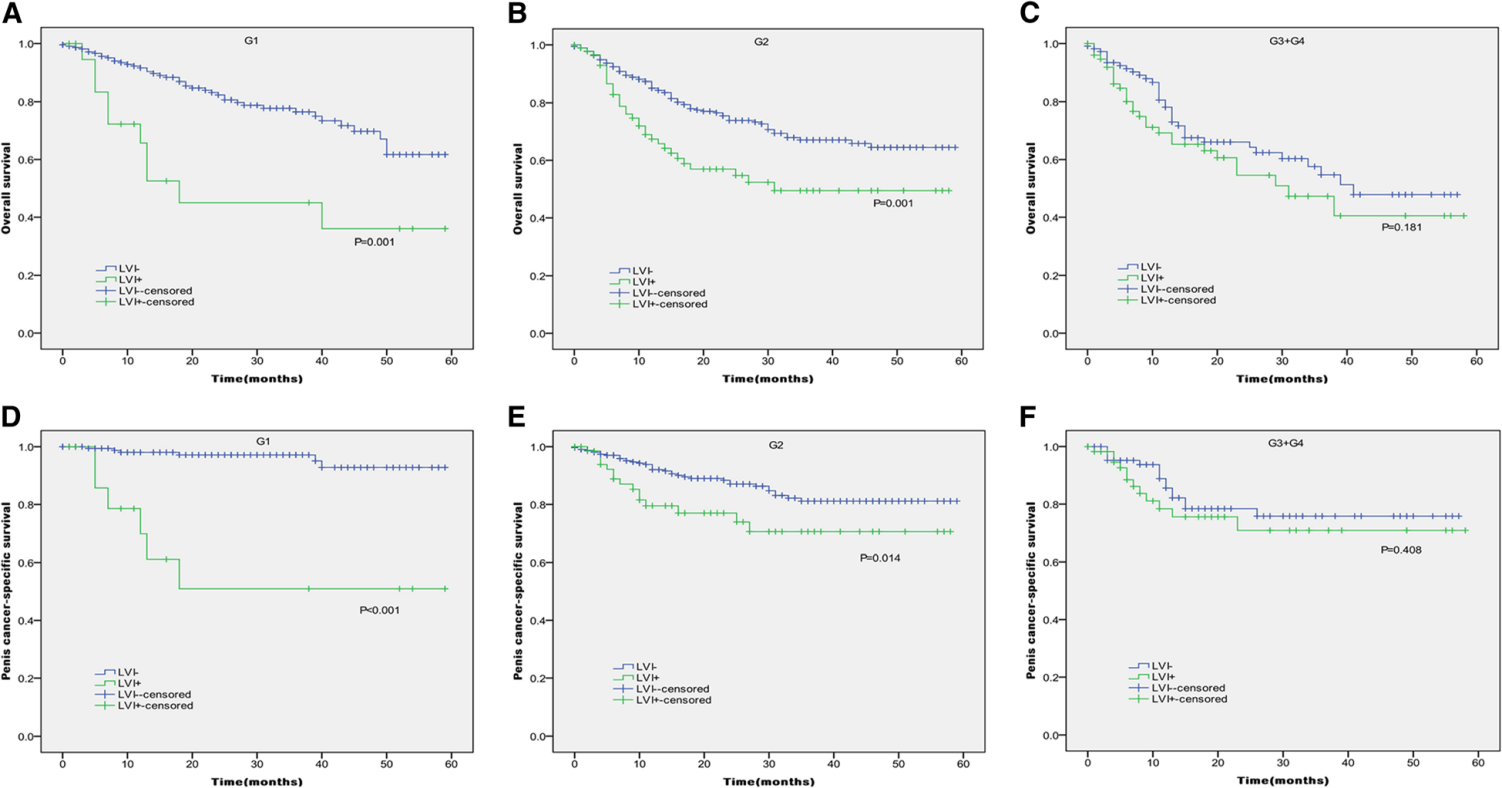
Kaplan-Meier analyses of overall survival (**a**, **b**, **c**) and penile carcinoma-specific survival(**d**, **e**, **f**) within each grade in patients treated with surgery stratified by LVI status

**Fig. 5:**
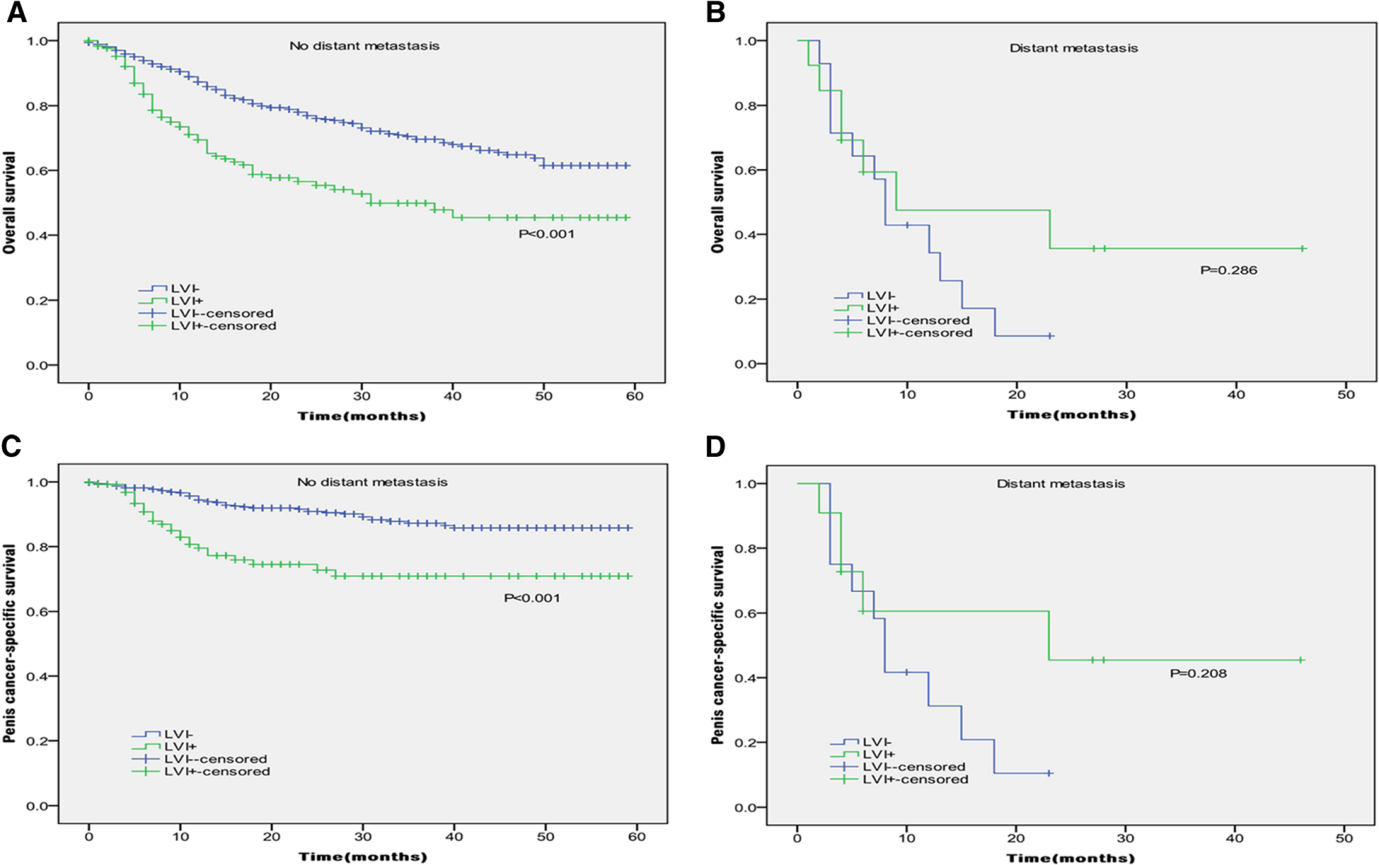
Kaplan-Meier analyses of overall survival (**a**, **b**) and penile carcinoma-specific survival (**c**, **d**) within no distant metastasis and distant metastasis in patients treated with surgery stratified by LVI status

**Fig. 6:**
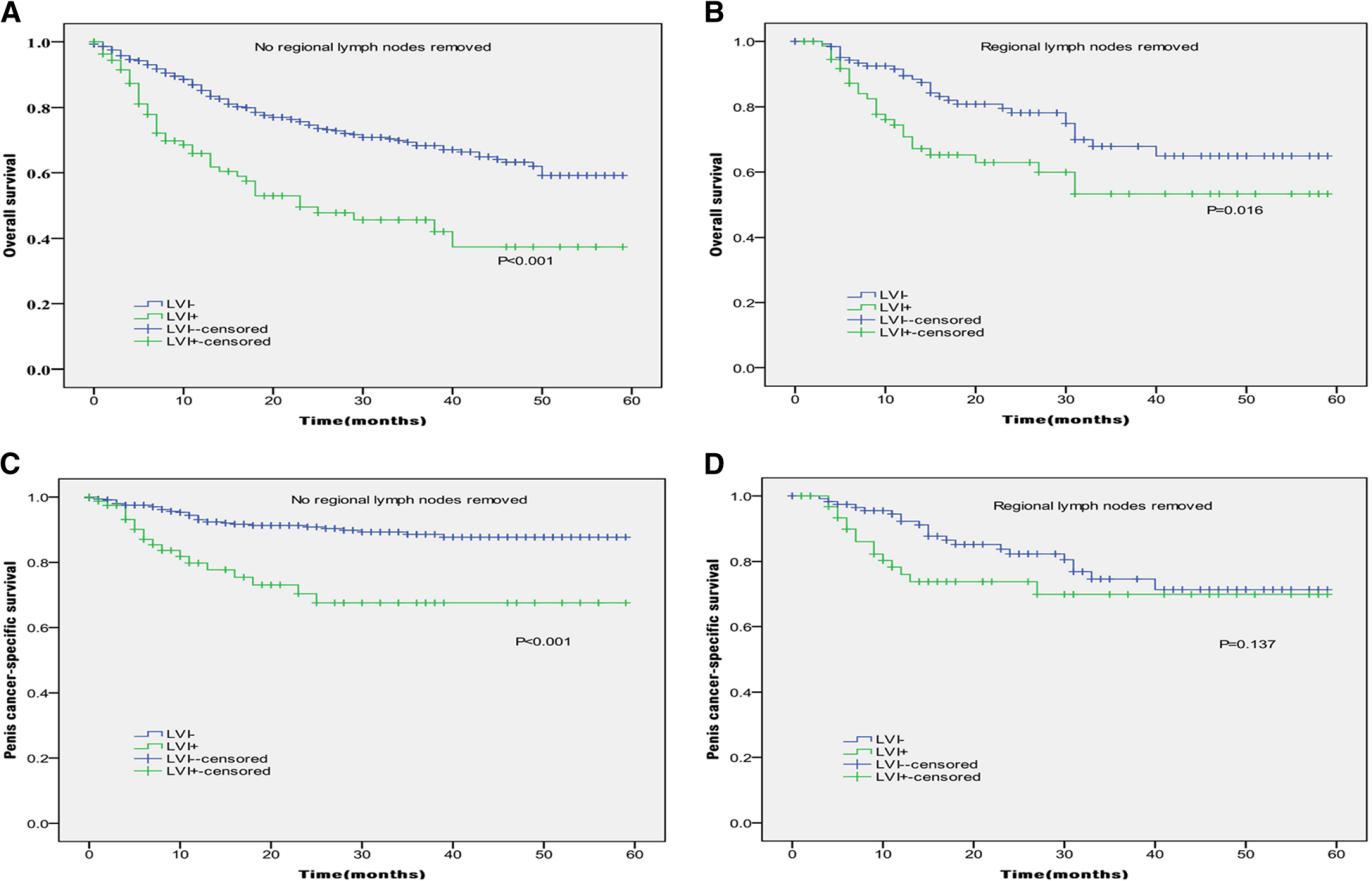
Kaplan-Meier analyses of overall survival (**a**, **b**) and penile carcinoma-specific survival (**c**, **d**) within no regional lymph nodes removed and regional lymph nodes removed in patients treated with surgery stratified by LVI status

**Fig. 7:**
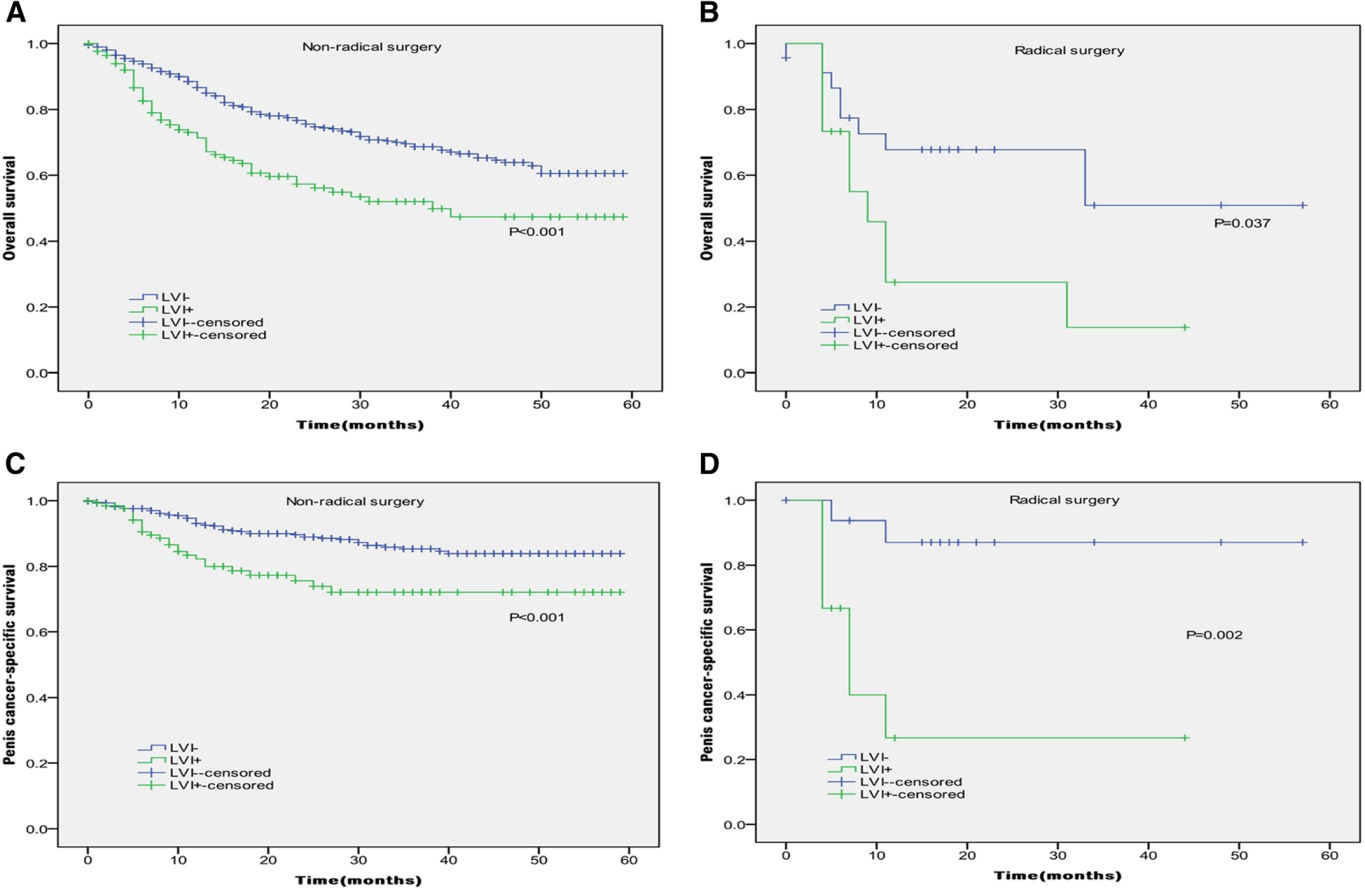
Kaplan-Meier analyses of overall survival (**a**, **b**) and penile carcinoma-specific survival (**c**, **d**) within non-radical surgery and radical surgery in patients treated with surgery stratified by LVI status

In Kaplan-Meier analyses, patients with the presence of LVI had significantly lower OS and PCSS than those with the absence of LVI (*P* < 0.001 for both, Table 2 and Fig. 1). The presence of LVI was also significantly associated with poorer OS and worse PCSS in patients with Tx + Ta + T1 stage (*P* = 0.007, *P* < 0.001, Table 2 and Fig. 2), N0 stage (P < 0.001, *P* = 0.040, Table 2 and Fig. 3), grade 1 (*P* = 0.001, P < 0.001, Table 2 and Fig. 4), grade 2 (P = 0.001, *P* = 0.014, Table 2 and Fig. 4), no distant metastasis (P < 0.001 for both, Table 2 and Fig. 5), no regional lymph nodes removed (P < 0.001 for both, Table 2 and Fig. 6), Non-radical surgery (P < 0.001 for both, Table 2 and Fig. 7) and radical surgery(*P* = 0.037, *P* = 0.002, Table 2 and Fig. 7). Moreover, the presence of LVI was significantly associated with poorer OS in patients with T2 stage (*P* = 0.011, Table 2 and Fig. 2) and regional lymph nodes removed (*P* = 0.016, Table 2 and Fig. 6). The 3-year survival was shown in Table 2.

**Table 2:** Overall survival and penis cancer-specific survival estimates with clinicopathological characteristics according to LVI

	Overall survival,%		Penile carcinoma-specific survival,%	
Group	3-Year Probability (SEM)	*P*	3-Year Probability (SEM)	*P*
All patients		**< 0.001**		**< 0.001**
LVI absent	68.1(2.3)		85.3(1.9)	
LVI present	48.6(4.6)		68.5(4.8)	
T stage				
Tx + Ta + T1		**0.007**		**< 0.001**
LVI absent	73.3(2.9)		90.7(2.0)	
LVI present	53.7(9.6)		65.4(10.3)	
T2		**0.011**		0.163
LVI absent	66.3(4.5)		80.4(4.5)	
LVI present	42.1(8.8)		75.4(8.3)	
T3		0.368		0.584
LVI absent	48.0(7.2)		69.9(7.0)	
LVI present	54.1(6.4)		69.0(7.0)	
T4		0.067		0.207
LVI absent	66.7(20.8)		85.7(11.7)	
LVI present	–		–	
Lymph nodes status				
Nx		0.826		0.515
LVI absent	44.5(17.7)		88.9(10.5)	
LVI present	57.1(18.7)		66.7(19.2)	
N0		**< 0.001**		**0.040**
LVI absent	72.9(2.4)		91.0(1.7)	
LVI present	56.7(6.0)		84.3(5.0)	
N1-N3		0.145		0.227
LVI absent	44.4(6.5)		54.7(7.2)	
LVI present	35.9(7.5)		48.1(8.6)	
Grade				
G1		**0.001**		**< 0.001**
LVI absent	76.4(3.7)		97.1(1.4)	
LVI present	45.0(12.7)		50.9(14.8)	
G2		**0.001**		**0.014**
LVI absent	67.0(3.2)		81.1(3.0)	
LVI present	49.5(6.6)		70.6(6.8)	
G3 + G4		0.181		0.408
LVI absent	54.7(6.4)		75.8(5.7)	
LVI present	47.3(7.8)		70.9(7.7)	
Distant metastasis				
No		**< 0.001**		**< 0.001**
LVI absent	69.6(2.3)		87.2(1.9)	
LVI present	49.9(4.8)		70.8(4.9)	
Yes		0.286		0.208
LVI absent	–		–	
LVI present	35.6(15.6)		45.5(17.6)	
Regional lymph nodes removed				
No		**< 0.001**		**< 0.001**
LVI absent	68.3(2.5)		88.6(1.9)	
LVI present	45.6(5.9)		67.6(6.7)	
Yes		**0.016**		0.137
LVI absent	67.9(5.4)		74.6(5.2)	
LVI present	53.3(7.4)		69.9(6.9)	
Surgery				
Non-radical surgery		**< 0.001**		**< 0.001**
LVI absent	68.7(2.3)		85.4(2.0)	
LVI present	52.0(4.8)		72.1(4.9)	
Radical surgery		**0.037**		**0.002**
LVI absent	50.8(16.5)		87.1(8.6)	
LVI present	13.7(11.8)		26.7(15.6)	

*LVI* lymphovascular invasion; *SCCP* squamous cell carcinoma of the penis; *SEM* standard error of mean

Significant values in bold, “-” = no data

The associations of clinicopathological variables with OS and PCSS are shown in Table 3. Univariate analyses found that LVI, grade, T stage, lymph nodes status, distant metastasis, regional lymph nodes removed, and surgery were significantly associated with OS and PCSS. Furthermore, although the results showed that regional lymph nodes removed, which played an important role in the treatment of penile cancer, wasn’t associated with OS (*P* = 0.690), we also included it in multivariate analyses.

**Table 3:** Univariate analyses of overall survival and penis cancer-specific survival

Variables	Overall survival			Penile carcinoma-specific survival		
	Hazard Ratio	95% CI	*P*	Hazard Ratio	95% CI	*P*
Age	1.556	0.962–2.517	0.071	0.796	0.450–1.407	0.432
T stage			**< 0.001**			**< 0.001**
Tx + Ta + T1		Referent	–		Referent	–
T2	1.543	1.134–2.100	**0.006**	1.827	1.086–3.074	**0.023**
T3	2.200	1.601–3.023	**< 0.001**	3.278	1.982–5.422	**< 0.001**
T4	2.976	1.208–7.333	**0.018**	5.687	1.730–18.701	**0.004**
Lymph nodes status			**< 0.001**			**< 0.001**
Nx		Referent	–		Referent	–
N0	0.367	0.180–0.747	**0.006**	0.226	0.070–0.733	**0.013**
N1-N3	0.900	0.434–1.866	0.777	1.384	0.431–4.444	0.585
Grade			**0.001**			**0.001**
G1		Referent	–		Referent	–
G2	1.332	0.961–1.847	0.085	2.367	1.263–4.435	**0.007**
G3 + G4	2.020	1.402–2.912	**< 0.001**	3.653	1.849–7.217	**< 0.001**
Distant metastasis	4.874	3.038–7.818	**< 0.001**	10.161	5.802–17.794	**< 0.001**
regional lymph nodes removed	0.940	0.695–1.272	0.690	1.832	1.204–2.790	**0.005**
Surgery	2.390	1.476–3.870	**< 0.001**	3.304	1.657–6.590	**0.001**
LVI	2.078	1.576–2.741	**< 0.001**	2.741	1.786–4.205	**< 0.001**

*SCCP* squamous cell carcinoma of the penis; *LVI* lymphovascular invasion; *CI* confidence intervals

Significant values in bold, “-” = no data

The multivariate Cox proportional hazards analyses, for prediction of OS and PCSS in patients with SCCP, who received surgery, was shown in Table 4. The results indicated that the presence of LVI in SCCP was an independent predictor for decreased OS (hazard ratio 1.403, *P* = 0.039), after adjusting for T stage, grade, lymph nodes status, distant metastasis, regional lymph nodes removed, and surgery. However, LVI was not found to be significantly associated with PCSS (hazard ratio 1.324, *P* = 0.277). Furthermore, lymph node status (*P* < 0.001 for both) and distant metastasis (hazard ratio 1.796, *P* = 0.035; hazard ratio 2.938, *P* = 0.002) were also significantly independently associated with poor OS and PCSS. T2 stage (hazard ratio 1.405, *P* = 0.040), T3 stage (hazard ratio 1.528, *P* = 0.028), G3 + G4 (hazard ratio 1.484, *P* = 0.049), regional lymph nodes removed (hazard ratio 0.457, P < 0.001) and surgery (hazard ratio 1.768, P = 0.028) were also associated with poor OS according to this model.

**Table 4:** Multivariate Cox regression analyses predicting overall survival and penis cancer-specific survival

Variables	Overall survival			Penile carcinoma-specific survival		
	Hazard Ratio	95% CI	*P*	Hazard Ratio	95% CI	*P*
T stage			0.066			0.420
Tx + Ta + T1	1.000	Referent	–	1.000	Referent	–
T2	1.405	1.016–1.945	**0.040**	1.219	0.701–2.118	0.483
T3	1.528	1.046–2.230	**0.028**	1.302	0.709–2.391	0.395
T4	2.134	0.839–5.428	0.112	2.786	0.797–9.740	0.109
Lymph nodes status			**< 0.001**			**< 0.001**
Nx	1.000	Referent	–	1.000	Referent	–
N0	0.522	0.253–1.079	0.079	0.361	0.104–1.247	0.107
N1-N3	1.290	0.596–2.791	0.518	1.645	0.465–5.820	0.440
Grade			0.068			0.171
G1	1.000	Referent	–	1.000	Referent	–
G2	1.057	0.750–1.488	0.752	1.342	0.691–2.607	0.386
G3 + G4	1.484	1.002–2.197	**0.049**	1.913	0.924–3.959	0.080
Distant metastasis	1.796	1.042–3.095	**0.035**	2.938	1.484–5.815	**0.002**
regional lymph nodes removed	0.457	0.314–0.666	**< 0.001**	0.734	0.424–1.270	0.269
Surgery	1.768	1.063–2.940	**0.028**	1.992	0.947–4.192	0.069
LVI	1.403	1.017–1.936	**0.039**	1.324	0.798–2.198	0.277

*SCCP* squamous cell carcinoma of the penis; *LVI* lymphovascular invasion; *CI* confidence intervals

Significant values in bold

## Discussion

Although SCCP is a rare disease among men across the globe, it is a significant health problem in most of the developing countries. Owing to rarity, there is a paucity of data to help with clinical decision making regarding the treatment of SCCP. To the best of our knowledge, the present study is first large study including 891 patients with SCCP following surgery. The results of the present study demonstrated that the presence of LVI was the significant independent predictor of decreased OS in patients with SCCP following surgery.

Consistent with previous studies [1, 16–18], the present study also revealed that LVI was notably associated with metastases to lymph nodes. This finding supports the hypothesis that lymphatic vessel invasion precedes or occurs concurrently with lymph node metastasis [6]. Moreover, the presence of LVI could significantly reduce OS and PCSS in patients with N0 stage but not in the NI-N3 stage. It indicated that the status of LVI in patients with no clinically evident metastasis was a significant predictor of OS and PCSS. This study also revealed that the presence of LVI increased the risk of distant metastasis. Furthermore, it is well-known that the infiltration of tumor cells into lymphatics or vessels is a crucial step in tumor dissemination. And the presence of LVI was noticeably associated with poor outcome in lymph node-negative patients. As reported by recent studies [19], these findings revealed that LVI might be an important predictor not only of lymphatic but also the hematogenous spread of SCCP. Moreover, LVI was closely associated with T stage and tumor grade. Previous studies reported that 82.7% of the patients with SCCP, with invasive or poorly differentiated tumors, had lymph node metastases [20]. Recent studies also reported that tumor stage and poorly differentiated cancer were independent predictors of lymph node metastases in penile squamous cancer [17]. Thus, the present association study between LVI status and T stage or tumor grade further highlighted the significance of LVI as a predictor of SCCP.

An early study including 145 patients with penile cancer treated in São Paulo, Brazil, reported that lymphatic and venous embolizations were not significantly associated with disease-free and overall survival according to Kaplan-Meier analyses [16]. Conversely, the findings of this study demonstrated that the presence of LVI significantly reduced the OS and PCSS in patients with SCCP in univariate analyses. Moreover, we also determined that the patients with LVI exhibited a lower OS and PCSS than those without LVI for each clinicopathological characteristic (Tx + Ta + T1 stage, N0 stage, grade 1, grade 2, no distant metastasis), suggesting that LVI might play a crucial role in the prognosis of early SCCP.

Using multivariate Cox proportional hazards analyses, the study described a risk classification for men with SCCP. The results demonstrated that the presence of LVI in SCCP was an independent prognostic factor for OS but not for PCSS, after adjusting for T stage, grade, lymph nodes status, distant metastasis, regional lymph nodes removed, and surgery. These findings were similar to those reported by Costa et al. [19], their data showed that LVI was an independent predictor of recurrence-free survival but not of disease-specific survival in patients with penile carcinoma. In contrast, Liu et al. [21] reported that vascular or lymphatic invasion was not significantly associated with overall survival of patients with SCCP. The differences between the results of the present study and those reported by Liu et al. could be attributed to differences in the study population.

Furthermore, the study confirmed that patients who removed regional lymph nodes exhibited lower occurrence of SCCP with LVI than those who did not. Similarly, patients who received radical surgery showed a lower incidence of SCCP with LVI compared to those who did not. These results suggested that patients with positive LVI would be more likely to undergo radical surgery and lymphadenectomy. As reported by Guimarães et al. [22], amputation and regional lymphadenectomy were preferred treatment of choice for invasive penile cancer.

Although this study was carefully conducted, several limitations to this study do exist. First, the study population was only comprised of some patients from the United States. Moreover, this is a retrospective analysis of patients with SCCP, and it is difficult to organize large prospective studies to detect the role of LVI. Another limitation was the fact that our study was based on the covariates of cases recorded in the SEER database as we could not get the medical charts of each patient. Despite these limitations, this study was able to demonstrate the effectiveness of LVI as a crucial prognostic indicator for SCCP. However, further studies are needed to validate these results.

## Conclusions

In conclusion, LVI is significantly associated with infaust clinicopathological characteristics. It was also associated with increased risk of advanced T stage, high grade, lymph node metastasis and distant metastasis, and patients with the presence of LVI exhibited a worse OS and PCSS than those without the presence of LVI for each clinicopathological characteristic (Tx + Ta + T1 stage, N0 stage, grade 1, grade 2, no distant metastasis). Moreover, our data suggested that LVI was an independent predictor of decreased OS in patients with SCCP following surgery. Taken together, the results suggested that the LVI status might be a crucial prognostic indicator for overall survival in patients with SCCP following surgery. Besides, these findings may guide in the surveillance of therapeutic modalities for penile cancer.

## Abbreviations

AJCC: American Joint Committee on Cancer
CI: Confidence intervals
HR: Hazard ratio
LVI: Lymphovascular Invasion
OS: Overall survival
PCSS: Penile carcinoma-specific survival
SCCP: Squamous cell carcinoma of the penis
SEER: The Surveillance, Epidemiology, and End Results database
